# Change in practice patterns over time for endocrine therapy and radiation therapy in women with breast cancer age 65 and older

**DOI:** 10.1007/s10549-026-07977-7

**Published:** 2026-05-20

**Authors:** Aubrey Stickland, Xueting Tao, Kelley Kidwell, N. Lynn Henry

**Affiliations:** 1 Department of Internal Medicine, University of Michigan Medical School, Ann Arbor, USA; 2 Department of Biostatistics, University of Michigan School of Public Health, Ann Arbor, USA; 3 Rogel Cancer Center, University of Michigan, Ann Arbor, USA; 4 1500 E. Medical Center Dr, Ann Arbor, MI 48109 USA

**Keywords:** Breast cancer, Guideline implementation, Older women, Endocrine therapy, Radiation therapy

## Abstract

**Purpose:**

In 2017, the National Comprehensive Cancer Center (NCCN) recommended omitting radiation therapy (RT) for women aged 70 + with stage 1, hormone receptor-positive breast cancer after lumpectomy. However, the optimal treatment remains uncertain given challenges with delivering endocrine therapy (ET) and RT.

**Methods:**

Females with hormone receptor positive, HER2 negative, clinically node negative breast cancer aged 65 + diagnosed 2012–2021 who underwent lumpectomy with or without axillary surgery at a single institution were included. The treatment groups of ET only, RT only, both ET and RT (ET/RT), and neither ET nor RT were compared by patient age, year of diagnosis, and recurrence rates. Fisher’s Exact Test was used to compare treatment groups, and competing risk analysis was used to examine recurrence rates.

**Results:**

Of 383 patients analyzed, 28% received ET only, 8% RT only, 53% ET/RT, and 10% neither ET nor RT. Over time, fewer participants received ET/RT or RT alone and more participants received ET alone or neither ET nor RT (*p*=.006). Compared to those treated with ET/RT, those treated with neither ET nor RT (hazard ratio [HR] 10.60 [95% CI 4.13–27.21]) and with ET alone (HR 3.02 [95% CI 1.28–7.16]) had higher risk of disease recurrence.

**Conclusions:**

Fewer participants received ET/RT or RT alone after the NCCN guideline publication. However, these cohorts had lower recurrence risks than those treated with ET only and neither ET nor RT. These results support consideration of larger prospective studies evaluating RT alone following lumpectomy in this population.

## Introduction

The mainstay of treatment for hormone receptor-positive, HER2-negative breast cancer for localized node-negative disease has traditionally been breast-conserving surgery followed by radiation therapy (RT) and endocrine therapy (ET). Treatment with ET has shown a 50% reduction in risk of recurrence compared to no ET when used for the recommended 5 year duration [1]. Similarly, the use of RT compared to no RT shows a 50% risk reduction in recurrence after lumpectomy [2]. Multiple studies have demonstrated a reduction in locoregional recurrence with the addition of RT to ET, compared to ET alone. In 2013, the Cancer and Leukemia Group B (CALGB) study C9343 showed a reduction in locoregional recurrence for women 70 years and older with the addition of RT to tamoxifen, but no change in overall survival or distant recurrence [3, 4]. The PRIME II trial demonstrated similar outcomes with a 8.6% absolute reduction in incidence of local recurrence for women 65 years and older who underwent RT compared to those who did not undergo RT [5]. However, these described benefits of RT on breast cancer outcomes were identified in clinical trial settings in patients with demonstrated or assumed good compliance with ET [3, 5]. 

There are many issues that may impact completion of both ET and RT. Many patients do not complete the recommended duration of ET because of treatment-emergent toxicity [6, 7]. In contrast, RT may be challenging for some patients to receive because of logistical challenges related to distance to treatment facility [8, 9] or financial considerations; [10] these barriers may be less of an issue with the advent of hypofractionated RT, ultra-hypofractionated RT, and accelerated partial breast irradiation (APBI) [11–14]. Additionally, some patients are concerned about potential acute and long-term toxicities of RT [15].

While the benefits of adding treatment with RT to adjuvant ET are well documented, there was a concern that given the relatively low absolute magnitude of benefit in patients with low-risk disease, some patients were being exposed to toxicity unnecessarily. In 2017, the National Comprehensive Cancer Network (NCCN) recommended consideration of omission of RT for women over age 70 with low risk, stage 1, hormone receptor-positive breast cancer after lumpectomy who will receive ET, based on lack of survival data from multiple studies of RT omission [16]. Our goal for this analysis was to analyze practice patterns over time at a single institution to determine whether administered treatments changed after the guidelines were published, as well as to examine associations between delivered treatments and breast cancer recurrence.

## Methods

**Fig. 1:**
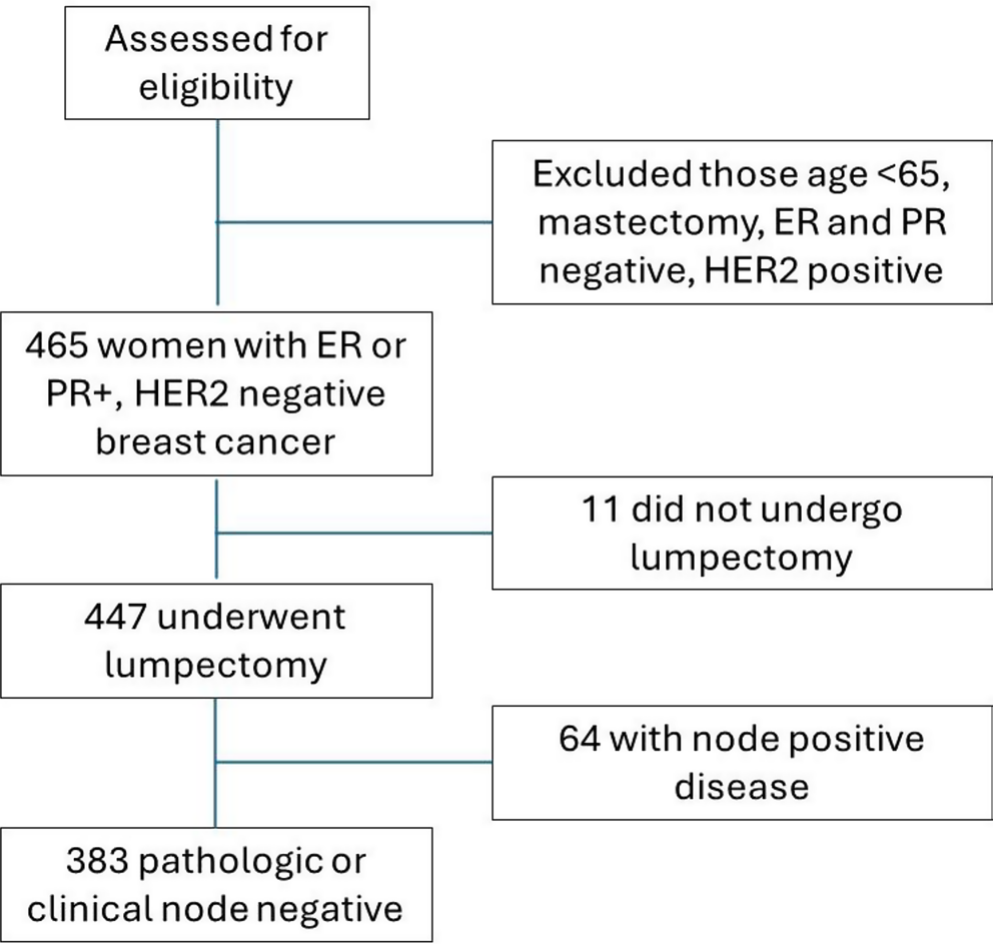
CONSORT diagram depicting selection of analyzed cohort. ER: estrogen receptor; PR: progesterone receptor

An Institutional Review Board (HUM00169453) exemption was obtained at the University of Michigan. DataDirect was used to identify potential patients in the Michigan Medicine Cancer Registry from years 2012–2021 by querying the electronic medical record to identify those in the registry who were aged 65 years or older and had stage 1 or 2 disease. Patients who were female, aged 65 or older, with hormone receptor positive, HER2 negative, clinically node negative breast cancer who underwent lumpectomy with or without sentinel lymph node biopsy were eligible for inclusion. Patients with cancers that were both estrogen and progesterone receptor non-overexpressing, who underwent mastectomy, or who had pathologic evidence of lymph node involvement were excluded (Fig. 1). We omitted patients diagnosed in 2020 as treatment patterns may have been altered due to the global pandemic. Additional data elements, including demographic data, diagnostic and treatment regimens and dates, and breast cancer events (contralateral breast primary, locoregional recurrence, and distant recurrence) were manually abstracted from the electronic medical record (EMR). Data was stored in the secure REDCap electronic data capture system [17]. 

Treatment groups were defined as receipt of ET alone, RT alone, both ET and RT, and neither ET nor RT. The groups were compared by patient age at diagnosis, year of diagnosis, duration of therapy, and breast cancer events using Fisher’s exact tests. The Fine–Gray subdistribution hazard model was used to examine discontinuation of treatment and breast cancer events over time between treatment groups, accounting for the competing risk of death. Predicted cumulative incidence functions (CIFs) for recurrence were obtained from the Fine–Gray competing risks regression model fitted with the cmprsk R package. Model-based predicted CIF curves were generated for each therapy group and plotted as step functions over time. The duration of ET was censored by the last visit to Michigan Medicine according to the EMR to account for lost to follow-up and time since diagnosis.

## Results

### Patient baseline characteristics

There were 383 patients who met eligibility criteria included in the analysis. Baseline characteristics are provided in Table 1. The majority of patients were white (355, 93%) and non-Hispanic (356, 93%). 70% of patients underwent sentinel lymph node biopsy (267, 70%); few received chemotherapy (22, 5.7%). Median follow-up for the entire cohort was 5 years (range 3.1-8).

**Table 1:** Demographic data for included participants

Characteristic	Total (*n* = 383)	Both Endocrine Therapy and Radiation Therapy (*n* = 203)	Endocrine Therapy Only (*n* = 108)	Radiation Therapy Only (*n* = 32)	Neither Endocrine Therapy nor Radiation Therapy (*n* = 40)
**Race**					
Asian	4 (1%)	4 (2%)	0 (0%)	0 (0%)	0 (0%)
Black or African American	14 (3.7%)	7 (3.4%)	5 (4.6%)	0 (0%)	2 (5%)
Native Hawaiian or Other Pacific Islander	1 (0.3%)	1 (0.5%)	0 (0%)	0 (0%)	0 (0%)
White	355 (93%)	186 (92%)	101 (94%)	31 (97%)	37 (93%)
Other	2 (0.5%)	1 (0.5%)	0 (0%)	1 (3.1%)	0 (0%)
Unknown	7 (1.8%)	4 (2%)	2 (1.9%)	0 (0%)	1 (2.5%)
**Ethnicity**					
Hispanic or Latino	2 (0.5%)	2 (1.0%)	0 (0%)	0 (0%)	0 (0%)
Non-Hispanic or Latino	356 (93%)	189 (93%)	99 (92%)	30 (94%)	38 (95%)
Unknown	25 (6.5%)	12 (5.9%)	9 (8.3%)	2 (6.3%)	2 (5%)
**Histology**					
Invasive ductal carcinoma	306 (79.6%)	157 (77.5%)	92 (85%)	25 (78%)	32 (80%)
Invasive lobular carcinoma	66 (17%)	44 (22%)	14 (13%)	3 (9.4%)	5 (13%)
Micropapillary carcinoma	1 (0.3%)	0 (0%)	0 (0%)	1 (3.1%)	0 (0%)
Mucinous adenocarcinoma	6 (1.6%)	2 (1%)	2 (1.9%)	0 (0%)	2 (5%)
Tubular adenocarcinoma	4 (1.0%)	0 (0%)	0 (0%)	3 (9.4%)	1 (2.5%)
**BMI**					
Mean (SD)	29.7 (6.5)	30.1 (6.4)	30.3 (6.9)	28.1 (5.9)	28.0 (6.3)
**Chemotherapy**	22 (5.7%)	21 (10%)	1 (0.9%)	0 (0%)	0 (0%)
**Axillary surgery** ^**A**^	267 (70%)	176 (87%)	56 (52%)	20 (63%)	15 (38%)
**Tumor Sizes**					
T1mi	3 (0.8%)	1 (0.5%)	1 (0.9%)	0 (0%)	1 (2.5%)
T1a	48 (13%)	23 (11%)	8 (7.4%)	8 (25%)	9 (23%)
T1b	108 (28%)	56 (28%)	30 (28%)	12 (38%)	10 (25%)
T1c	161 (42%)	84 (41%)	56 (52%)	11 (34%)	10 (25%)
T2	62 (16%)	38 (19%)	13 (12%)	1 (3.1%)	10 (25%)
T3	1 (0.3%)	1 (0.5%)	0 (0%)	0 (0%)	0 (0%)
**Estrogen Receptor**					
Positive (> 10%)	383 (100%)	203 (100%)	108 (100%)	32 (100%)	40 (100%)
**Progesterone Receptor**					
Negative	39 (10%)	22 (11%)	13 (12%)	2 (6.3%)	2 (5%)
Positive (> 10%)	314 (82%)	161 (79%)	87 (81%)	29 (91%)	37 (93%)
Weakly positive (< = 10%)	30 (7.8%)	20 (10%)	8 (7.4%)	1 (3.1%)	1 (2.5%)

^A^All but one participant who underwent axillary surgery had a sentinel lymph node biopsy; the other underwent axillary lymph node dissection and subsequently received both endocrine therapy and radiotherapy

### Practice patterns for ET and RT

Across all analyzed years, just over half of women received both ET and RT (203 [53%]), while 108 (28%) received ET only, 32 (8.4%) were treated with RT only, and 40 (10.4%) received neither ET nor RT. Of the 311 patients who received any ET, 252 (81%) received anastrozole at some point during their course of therapy, whereas 59 (19%) received at least some tamoxifen. Use of ET and RT changed over time in this patient population (*p*=.01, Fig. 2A). In both 2015–2016 and 2019–2021, more women received ET alone and fewer women either received both ET and RT or received RT alone compared to 2012–2013. Receipt of neither ET nor RT also numerically increased over time.

**Fig. 2a:**
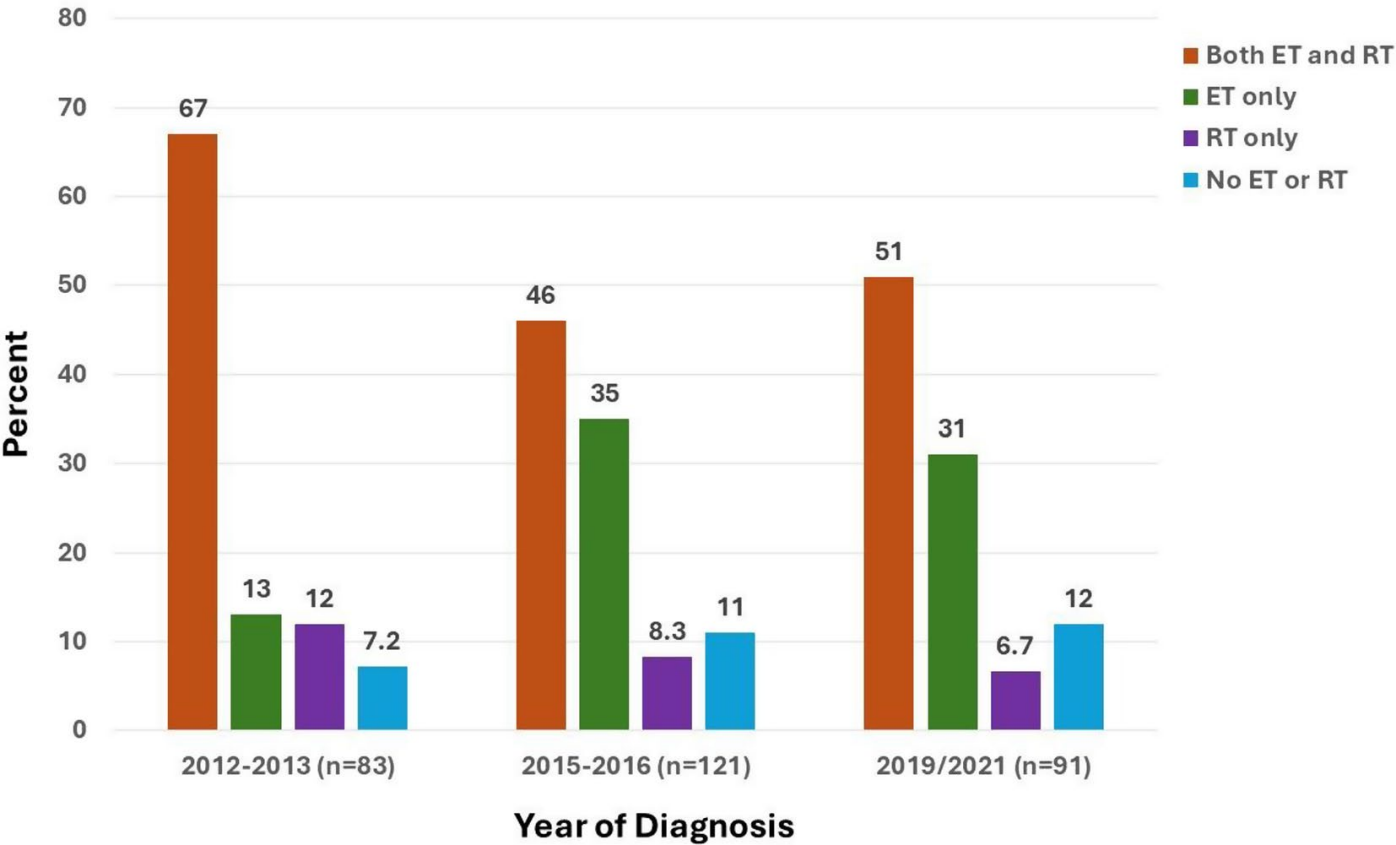
Treatment received by year of diagnosis. Percentage of patients treated with both endocrine therapy (ET) and radiotherapy (RT) (orange), ET only (green), RT only (purple), and neither ET nor RT (blue) are shown

Figure 2a Treatment received by year of diagnosis. Percentage of patients treated with both endocrine therapy (ET) and radiotherapy (RT) (orange), ET only (green), RT only (purple), and neither ET nor RT (blue) are shown by year of diagnosis.

Use of ET and RT also varied by age in this patient population (*p*<.001, Fig. 2B). Women aged less than 70 were more likely to receive both ET and RT compared to older women. In contrast, receipt of ET only and neither ET nor RT both increased with increasing age.

**Fig. 2b:**
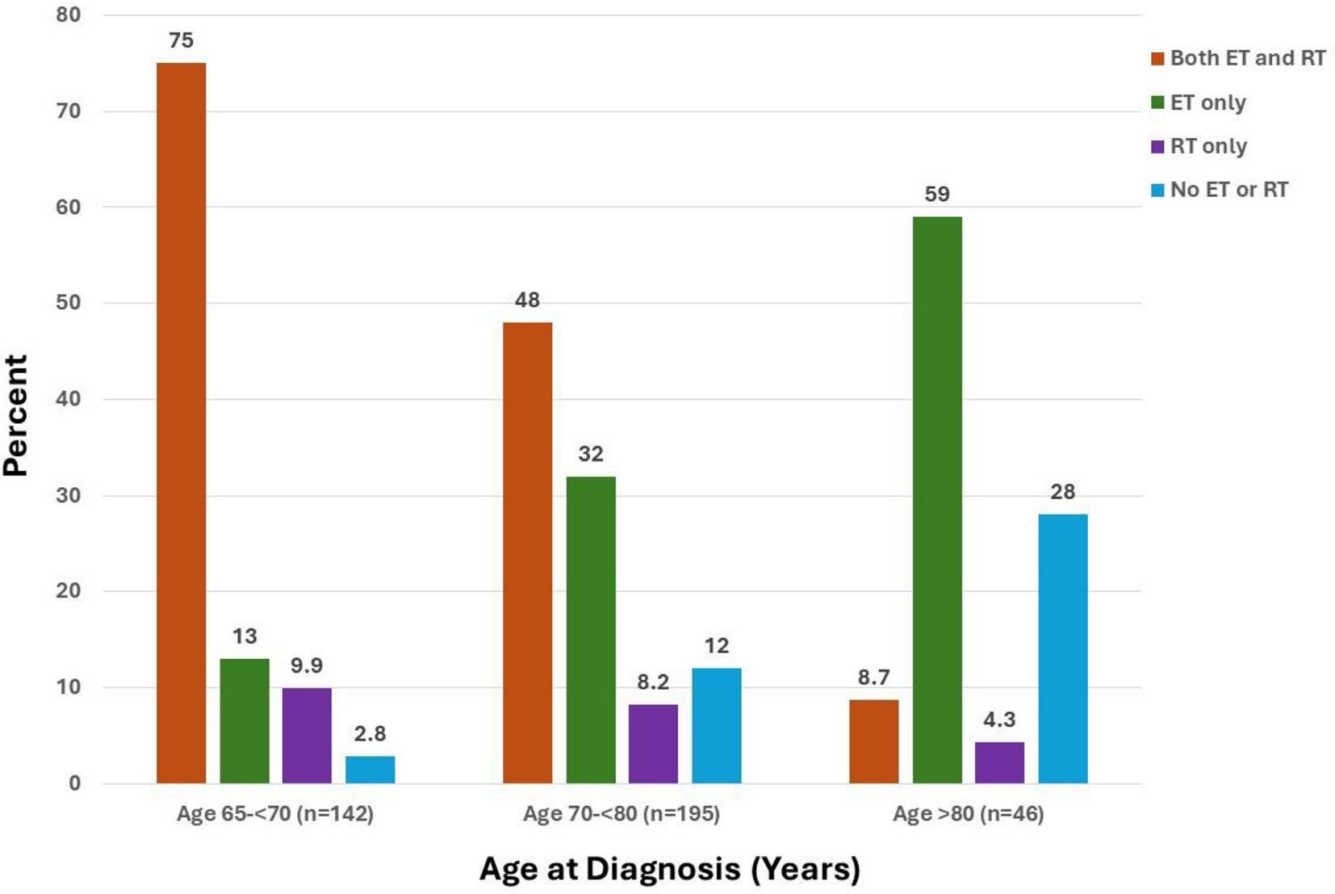
Treatment groups by age at diagnosis. Percentage of patients treated with both endocrine therapy (ET) and radiotherapy (RT) (orange), ET only (green), RT only (purple), and neither ET nor RT (blue) are shown by age at diagnosis

Of the analyzed cohort, 267 (70%) underwent axillary surgery. Those who had axillary surgery were more likely to receive both ET and RT (176 [66%]), compared to ET alone (56 [21%]), RT only (20 [7%]), and neither ET nor RT (15 [6%], *p* < .001). In contrast, women who did not undergo axillary surgery were numerically more likely to receive ET alone (52 [45%]), than both ET and RT (27 [23%]), neither ET or RT (25 [22%]), or RT alone (12 [10%]).

### Endocrine therapy duration

**Fig. 3:**
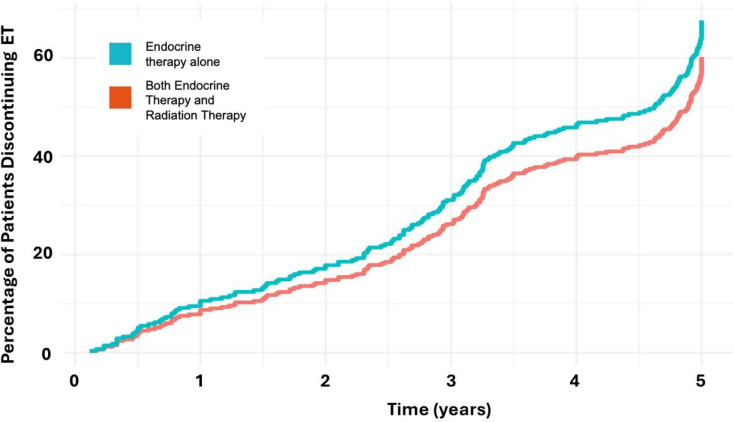
Cumulative incidence for endocrine therapy (ET) discontinuation. Orange = both ET and radiation therapy (RT), blue = ET alone

In the entire analyzed cohort, those treated with ET alone had a mean duration of ET therapy of 3.45 years (SD 1.56), compared to 3.68 years (SD 1.59) for those treated with both ET and RT. In a competing risk analysis, compared to patients treated with both ET and RT, patients treated with ET alone were 23% more likely to discontinue ET before 5 years (hazard ratio [HR] 1.23 (95% CI 0.92–1.64, *p*=.17; Fig. 3), although the difference was not statistically significant. When evaluated by year, average duration of ET in 2012–2013 was 3.77 years (SD 1.79), in 2015–2016 was 3.88 years (SD 1.66), and in 2019–2021 was 3.34 years (SD 1.39). When evaluated by age in the entire cohort, average duration of ET for women age 65–70 was 3.68 years (SD 1.64), for those age 70–80 was 3.66 years (SD 1.52), and for those age 80 and older was 3.01 years (SD 1.6).

### Recurrence

**Fig. 4:**
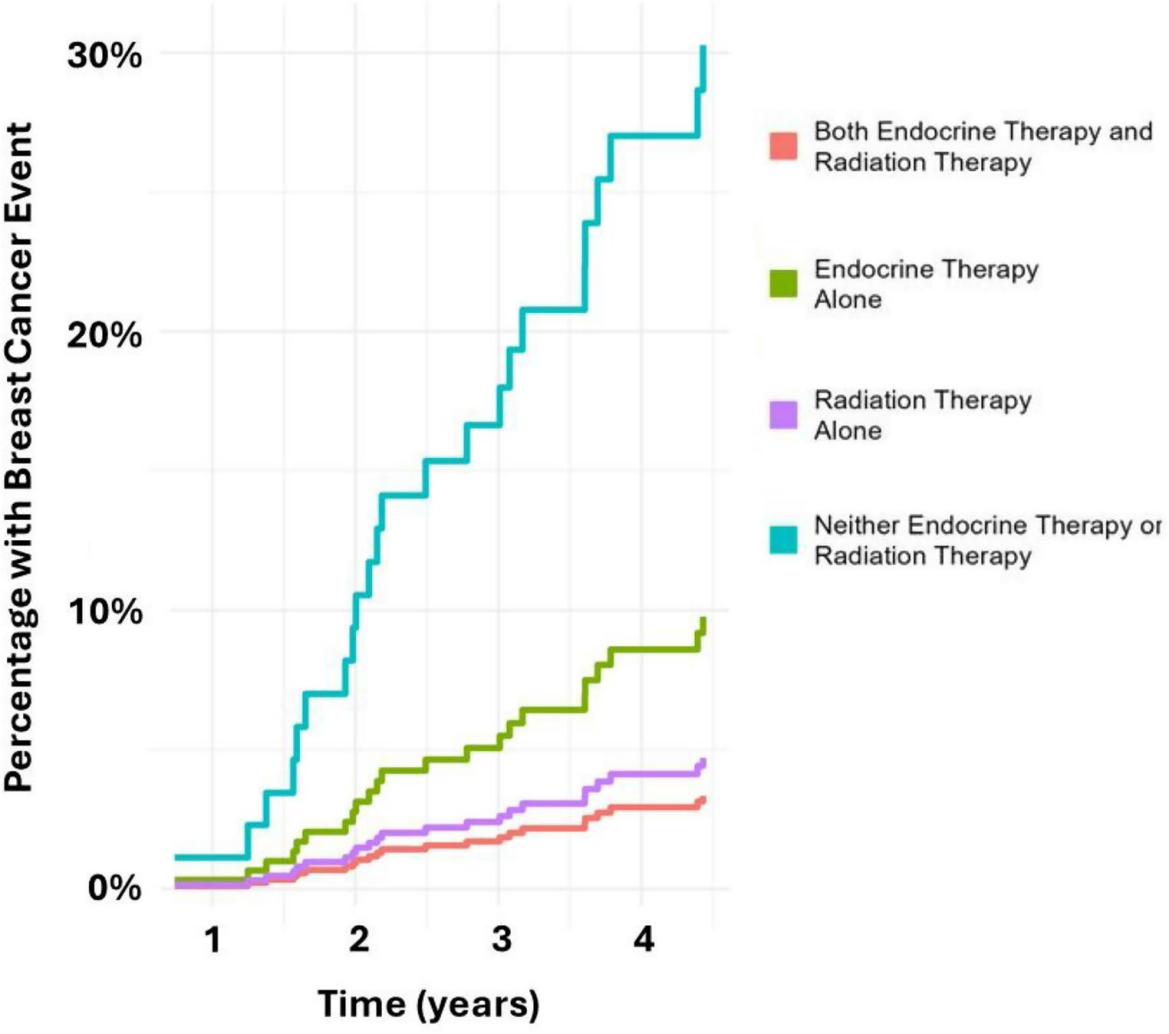
Cumulative incidence of breast cancer events (locoregional recurrence, distant recurrence, and new contralateral breast primary), assessed using a competing risks model to account for death, by treatment group. Endocrine therapy (ET) and radiotherapy (RT) (orange), ET only (green), RT only (purple), and neither ET nor RT (blue)

In the overall cohort, compared to patients treated with both ET and RT, both those treated with neither ET nor RT (HR 10.60 [95% CI 4.13–27.21]) and those treated with ET alone (HR 3.02 [95% CI 1.28–7.16]) had a higher risk of experiencing a breast cancer event (Fig. 4). In contrast, those treated with RT alone did not have a statistically significant higher risk of experiencing a breast cancer event (HR 1.42 [95% CI 0.30–6.70]), although the size of this subgroup was small.

Patients experienced locoregional and distant recurrence of disease, as well as new primary breast cancers in the contralateral breast. Of the 40 patients who received neither ET nor RT, 8 (20%) experienced locoregional recurrence and 2 (5%) developed cancer in the contralateral breast. Of the 108 patients treated with ET alone, 9 (8%) had locoregional and 2 (2%) had distant recurrence, and 1 (1%) was diagnosed with contralateral breast cancer. The two patients with disease recurrence (6%) of the 32 who had been treated with RT alone both had locoregional recurrence. Finally, of the 203 patients treated with both ET and RT, 4 (2%) had locoregional and 3 (1%) had distant recurrence, and 2 (1%) developed disease in the contralateral breast.

## Discussion

In this retrospective analysis of patients treated at a single institution over the past 13 years, we demonstrated a change in treatment patterns after the publication of NCCN guidelines recommending RT omission in patients with small, node negative, hormone receptor-positive, HER2-negative breast cancer. Notably, we found that since the new guidelines were released, more women have been receiving ET alone or receiving neither ET nor RT, and fewer women have been receiving either RT alone or both ET and RT.

The guidelines recommending omission of RT in this population are based on the assumption that patients will complete the full 5-year course of ET. It was surprising that the number of women receiving neither ET nor RT has been increasing. One possibility is a patient at lower risk of disease recurrence may be making the decision to forgo RT with her radiation oncologist prior to meeting with a medical oncologist, then hearing about the relatively low absolute benefit from ET and deciding to omit that treatment modality as well, without recognizing the implications of omitting both treatments.

Although fewer patients have received RT alone over time, there was a small numerical increase in the number of patients receiving RT plus ET in the most recent cohort compared to 2015–2016. It is possible that this uptick in RT use is due to increased acceptability of hypofractionated or APBI regimens. There is increasing recognition of the premature discontinuation of ET prior to the recommended 5–10 years; based on our data, the average total duration of ET across all cohorts is less than 4 years, and was only 3.34 years in the 2019–2021 cohort. A systematic review of 29 studies showed an adherence rate of endocrine therapy of 41–72% while early discontinuation ranged from 31 to 73% [18]. Studies have shown an initial severe side effect or the development of a side effect during the follow-up period increases the risk for early discontinuation [19–23]. The most common side effects women develop on ET are vasomotor symptoms, musculoskeletal symptoms, fatigue and insomnia [7, 23]. Therefore, now that there are shorter, more accessible RT regimen options available, some patients may be opting to receive radiotherapy in case they are unable to tolerate ET.

There is concern that taking less than the recommended duration of ET in the absence of RT will result in increased likelihood of breast cancer recurrence [24]. Our data are consistent with this concern. We found that the risk of breast cancer events for those treated with ET alone was higher compared to those who were treated with both ET and RT. Although we were not able to demonstrate a statistically significant difference in ET duration for those patients who received ET alone versus those who received both ET and RT, those who received ET alone were numerically more likely to stop taking ET prematurely. Additionally, it is possible that daily adherence to ET was also lower for those who received ET alone, although we were unable to assess adherence in this retrospective study.

One question is whether it is reasonable to recommend that patients take RT instead of ET, especially for those patients for whom there is concern about ET tolerability or persistence. This is especially relevant given the newer data demonstrating effectiveness of shorter RT treatment courses of 1–3 weeks, such as in the UK FAST FORWARD trial or APBI regimens, which may be more accessible for patients from tolerability, financial, and time toxicity perspectives [11–13]. 

Multiple trials have examined RT alone versus tamoxifen alone for treatment of tumors at lower risk of disease recurrence. Our finding of decreased risk of recurrence of disease with RT alone compared to ET alone is consistent with that of NSABP B-21. In the large NSABP trial, rates of ipsilateral breast tumor recurrence (IBTR) were 49% lower with RT plus placebo compared to tamoxifen alone in patients with tumors ≤ 1 cm. Additionally, in the NSABP trial the combination of tamoxifen and RT resulted in a 63% lower IBTR rate than RT plus placebo. Conversely, in the BASO II trial of 1135 patients with tumors < 2 cm and good prognostic features, after 10 years follow-up the rates of local recurrence in patients treated with tamoxifen alone or RT alone were similar (0.8% and 0.7% per year, respectively), in contrast to 2.2% per year for patients randomized to neither treatment [25]. These data are therefore mixed, and don’t clearly support omission of RT based on IBTR rates alone.

Researchers have previously used data from the SEER registry to examine omission of radiation therapy following publication of randomized phase 3 clinical trial results [26]. They noted incomplete uptake of radiotherapy omission, and questioned whether this may be due to substitution of conventionally fractionated whole breast radiotherapy with APBI. In our data we also identified a decrease over time in those receiving RT-based treatment approaches, albeit lower than expected. This limited decrease could be due in part to reluctance of patients or clinicians to completely de-escalate their treatment, especially for those younger than age 80 or with fewer comorbidities, and instead substitute conventionally fractionated radiotherapy with these shorter regimens. Prospective examination of reasons underlying treatment decision-making from both the patient and clinician perspective is important for understanding the nuances that impact care delivery.

A few trials have examined quality of life for patients receiving RT. The PRIME trial, which evaluated quality of life (QOL) [27] with RT versus omission of RT for older women on ET, did not show a difference in QOL between those who underwent RT versus those who omitted RT [28]. In the EUROPA trial, which is comparing RT versus ET in older patients, ET alone has been shown to be associated with a greater decline in QOL and physical functioning scores compared to RT alone; importantly, breast cancer recurrence data are not yet available [29]. Both of these results suggest that RT has minimal effects on a patient’s QOL, especially in the longer term, and may be more tolerable than ET. Additionally, it may be more practical for many women to tolerate 1–4 weeks of RT compared with 5 years of ET, especially if the long-term breast cancer outcomes are similar between the treatment regimens.

Another consideration is financial toxicity, especially for patients receiving RT; at the present time, the out-of-pocket cost of tamoxifen and aromatase inhibitor therapy is less of a concern because the medications are all generic and low cost. Cost of RT is significantly less expensive with hypofractionated or APBI regimens compared to conventionally fractionated whole breast regimens [30]. Patients can experience loss of income or job loss due to the need to receive daily radiotherapy treatments [10], although this also may be less of a concern with the adoption of shorter treatment regimens [14]. Finally, for patients who live farther from radiation oncology clinics there are concerns because of transportation issues that may limit access to care [31]. 

The strengths of our study include a large sample size with similar baseline characteristics between the different treatment groups. We also had a long follow-up period, with few patients lost to follow-up during the initial 5 years following breast cancer diagnosis. Limitations of this study include a lack of racial and ethnic diversity in our cohort. Additionally, the number of patients with recurrences was small, in part because of the favorable tumor characteristics for the included cohort, although this limits the statistical power for analysis of recurrence risk by treatment group. Furthermore, since the study was not randomized, there is likely bias related to choice of treatment that could influence recurrence rates in addition to the treatments themselves.

In summary, our study demonstrated practice pattern changes in our institution after the NCCN guideline changes, with more women omitting RT after lumpectomy. However, this carries the concern for increased recurrence rates, especially if patients also fail to complete the recommended 5 years of adjuvant ET. Our findings additionally suggest that RT alone may be a reasonable option for some older women with localized disease. This is especially true given the more recent changes in RT treatment approaches, including hypofractionation and ABPI, which may make radiation logistically more feasible for many patients. We await additional data from trials comparing modern endocrine therapies and radiotherapy regimens to fully understand the impact on disease outcomes as well as predictors of which patients may benefit from the different treatment approaches. 

## Data Availability

The datasets generated during and/or analyzed during the current study are not publicly available but are available from the corresponding author on reasonable request.
